# Supplementary figures and images for: Multi-tissue characterization of the constitutive heterochromatin proteome in Drosophila identifies a link between satellite DNA organization and transposon repression
Source: PLoS Biol. 2025 Jan 15;23(1):e3002984. doi: 10.1371/journal.pbio.3002984 (PMC11734925; doi:10.1371/journal.pbio.3002984)

Raw images for Fig. S1D

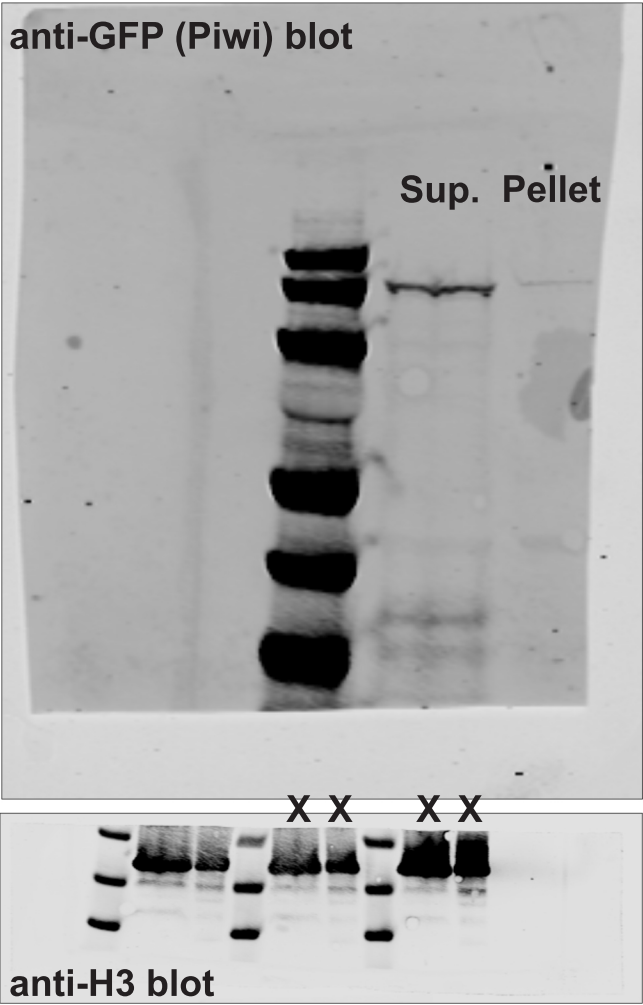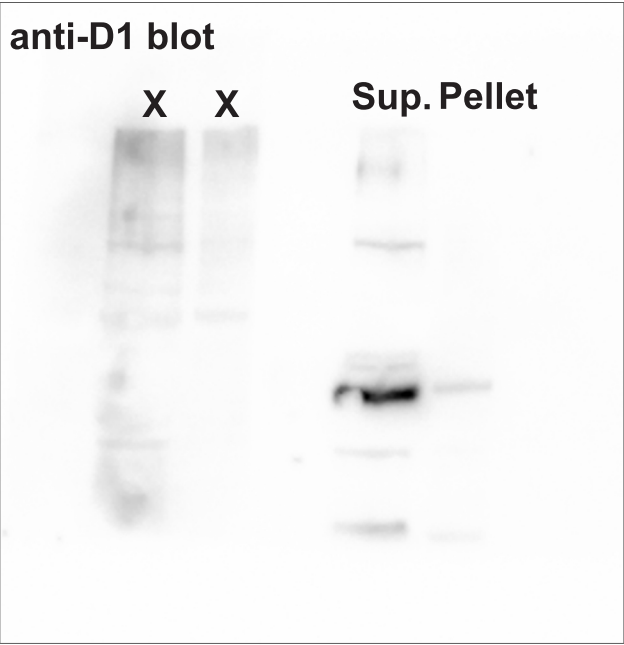

Raw images for Fig. S1E

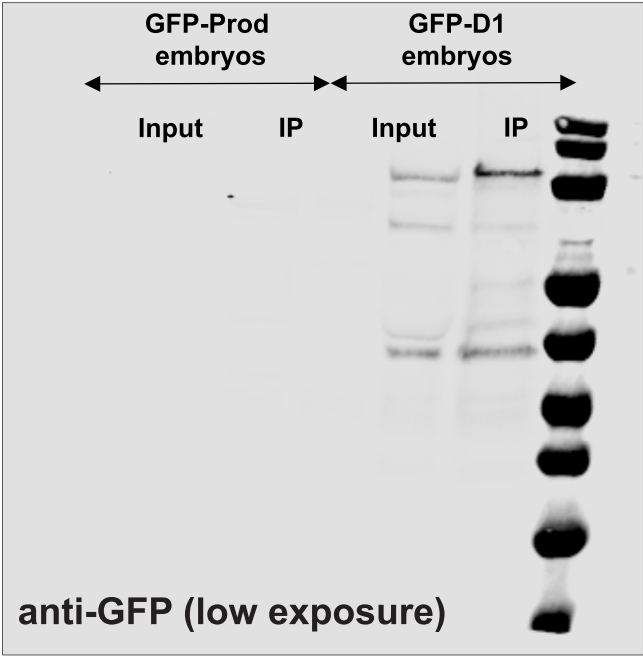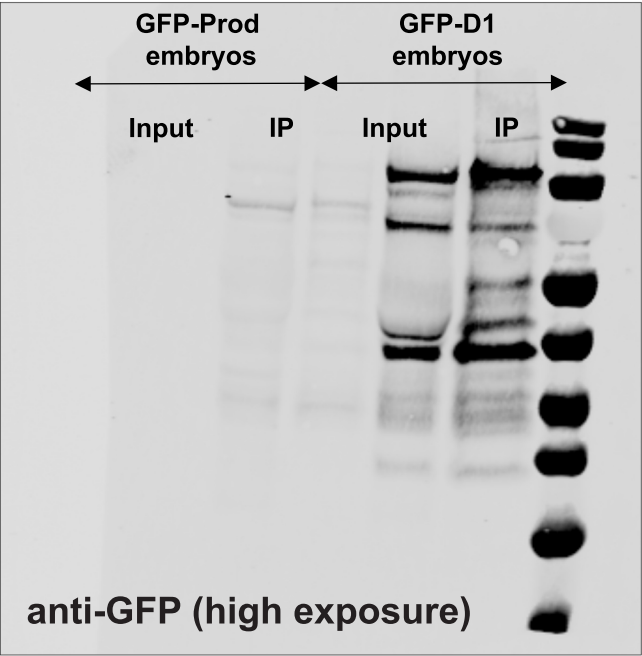

Raw images for Fig. S4B

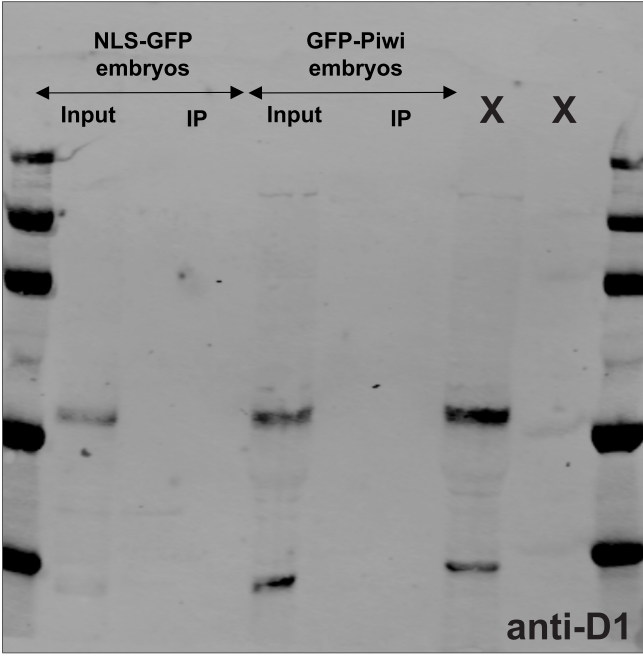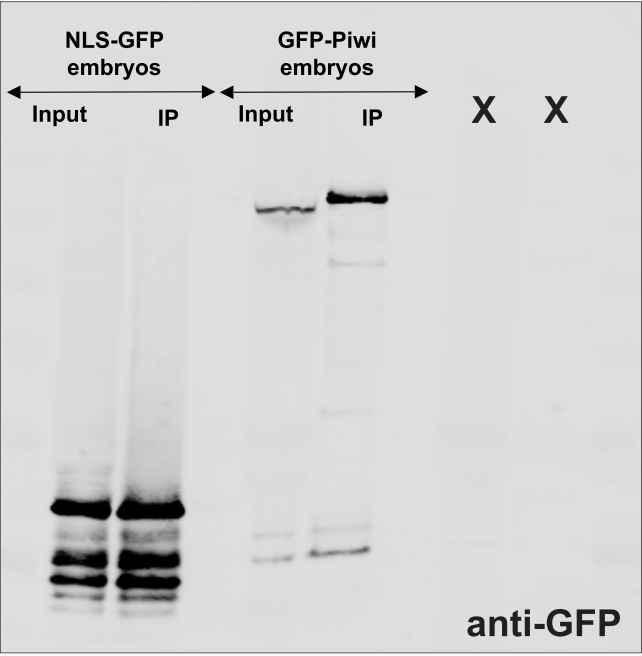

Supplement: S1 Raw Images — (PDF) [file pbio.3002984.s019.pdf]
