# Supplementary material for: Multi-tissue characterization of the constitutive heterochromatin proteome in Drosophila identifies a link between satellite DNA organization and transposon repression
Source: PLoS Biol. 2025 Jan 15;23(1):e3002984. doi: 10.1371/journal.pbio.3002984 (PMC11734925; doi:10.1371/journal.pbio.3002984)
Supplement: Attachment — Submitted filename: Response_v2.pdf [file pbio.3002984.s028.pdf]

Full Revision

Manuscript number: RC-2023-02080
Corresponding author(s): Madhav, Jagannathan

1. General Statements

We thank the reviewers for the overall positive and constructive comments on our manuscript. In this section, we have summarized the critical experiments that we have performed based the reviewers' suggestions. We are confident that we have addressed the key points raised by the reviewers and have substantially strengthened our manuscript. Please see section 2 for point-by-point responses to the reviewer comments.

The improvements to our revised manuscript can be classified into three main categories,

The link between the proteomic dataset and transposon expression. The reviewers pointed out that the connection between the D1/Prod interactions and the transposon expression phenotype was not completely clear in the original submission. In the revised manuscript, we have addressed this comment through changes in text (see point-by-point responses) and through experiments such as RNA-seq and small RNA seq. Essentially, our proteomic data identified associations between D1/Prod and TE repression proteins, specifically in the embryo. To better understand the functional relevance of these associations, we sought to deplete D1 in the early embryo. However, *D1* mutants (the progeny of heterozygous parents) contain maternal deposits during embryogenesis while protein depletion experiments in early embryos can be very challenging. On the other hand, the progeny of *D1* mutant females (F_2 *D1* mutant, Fig. S9A-C, revised manuscript) develop in the absence of both maternal and zygotic D1 protein. In the F_2 *D1* mutant, we observed a striking increase in transposon expression and agametic and atrophied ovaries. Moreover, the gonadal atrophy can be rescued by mutating the checkpoint kinase, *Chk2*, which mediates germ cell arrest in response to transposon mobilization (Fig. 6H, I, revised manuscript). Importantly, these deleterious phenotypes were not observed in the F_1 *D1* mutant, where maternally deposited D1 is present during embryogenesis (and potentially early larval development), or in the F_2 D1 heterozygote, where D1 is expressed during embryogenesis following zygotic genome activation (Fig. S9A-C, revised manuscript). Thus, our data suggest that embryogenesis (and potentially early larval development) as the time window during which D1 is critical for establishing heritable repression at TE loci. In the revised manuscript, we have improved our text in lines 383-391 to clarify that it was the associations with transposon repression proteins identified in embryos that guided

the logic and experimental design. In addition, we have restructured the introduction on TE repression as suggested by reviewer #2.

A full characterization of the role of the D1 satellite DNA-binding protein on transposon repression using RNA-seq, small RNA-seq and fertility assays. Although we did not observe an effect of D1/Prod depletion on commonly used transposon reporters (GFP-Burdock-lacZ, gypsy-lacZ), the reviewers were correct to point out that this does not necessarily rule out de-repression of other transposons. In addition, the reviewers suggested an additional genotype (F_2 *D1* heterozygote) to unambiguously determine whether TE expression in the F_2 *D1* mutant is caused by a maternal effect of the F_1 *D1* mutant i.e. impaired piRNA biogenesis due to RDC mislocalization. All of these experiments have been performed as suggested, have significantly improved our study and are explained in detail in the point-by-point responses below. Briefly, our data in the revised manuscript clearly demonstrate that flies lacking D1 during embryogenesis exhibit transposon expression, gonadal atrophy and sterility as adults. We further demonstrate that this gonadal atrophy can be rescued by mutating the checkpoint kinase, *Chk2*, which mediates germ cell arrest in response to transposon mobilization.

Improvements in the proteomic data analysis and presentation. As suggested by the reviewers, we have made efforts to further improve certain aspects of the proteomic data. Specifically, we have assessed our lysis conditions (450mM NaCl and benzonase) and the solubilization of our baits post-lysis. In Fig. S1D, we have blotted equivalent fractions of the soluble supernatant and insoluble pellet from GFP-Piwi embryos and show that both GFP-Piwi and D1 are largely solubilized following lysis. In Fig. S1E, we also show that our IP protocol works efficiently. In addition, we have added another biological replicate to the testis D1 IP-MS, and we now observe that the bait is enriched in 5/6 experiments (log_2FC between 4-12). The only instance in which we do not detect the bait by mass spectrometry is for GFP-Prod pulldown in embryos. Here, one reason could be relatively low expression of GFP-Prod in comparison to GFP-D1 (Fig. S1E), which may lead to technical difficulties in detecting peptides corresponding to Prod. However, we note that Prod pulldown co-purified proteins such as Bocks that were previously identified as Prod interactors from high-throughput studies (Giot et al. 2003; Guruharsha et al. 2011) while Prod also co-purified proteins known to associate with chromocenters such as Hcs (Reyes-Carmona et al. 2011) and Saf-B(Huo et al. 2020). In addition, the high concordance between D1 and Prod co-purified proteins from embryo lysates (Fig. 2A, C) suggest that the Prod IP from embryos is of reasonable quality. We have also improved our data analysis by incorporating an optimized imputation strategy and multiple testing corrections (Benjamini-Hochberg). Briefly, we noted that a lot of the variability in the proteomic data (and the resultant issues in the p-values) came from missing values in the control GFP pulldown and a noisy imputation strategy. For the revised analysis, we have used more

stringent data filtering and median imputation (see lines 652-665 in the materials and methods for details) and subsequently performed multiple testing corrections as suggested by reviewer #2 for most of our analyses. Finally, we have revised our text to clearly indicate which bait pulled down which protein in which tissue (lines 147-155). We have also highlighted and discussed bait-specific and tissue-specific interactions in the text (lines 157-173).

1. **Point-by-point description of the revisions**

This section is mandatory. Please insert a point-by-point reply describing the revisions that were already carried out and included in the transferred manuscript.

Reviewer #1 (Evidence, reproducibility, and clarity (Required)):

This is an interesting manuscript from the Jagannathan laboratory, which addresses the interaction proteome of two satellite DNA-binding proteins, D1 and Prod. To prevent a bias by different antibody affinities they use GFP-fusion proteins of D1 and Prod as baits and purified them using anti GFP nanobodies. They performed the purifications in three different tissues: embryo, ovary and GSC enriched testes. Across all experiments, they identified 500 proteins with surprisingly little overlap among tissues and between the two different baits. Based on the observed interaction of prod and D1 with members of the canonical piRNA pathway the authors hypothesized that both proteins might influence the expression of transposable elements. However, neither by specific RNAi alleles or mutants that lead to a down regulation of D1 and Prod in the gonadal soma nor in the germline did they find an effect on the repression of transposable elements. They also did not detect an effect of a removal of piRNA pathway proteins on satellite DNA clustering, which is mediated by Prod and D1. However, they do observe a mis-localisation of the piRNA biogenesis complex to an expanded satellite DNA in absence of D1, which presumably is the cause of a mis-regulation of transposable elements in the F2 generation.This is an interesting finding linking satellite DNA and transposable element regulation in the germline. However, I find the title profoundly misleading as the link between satellite DNA organization and transgenerational transposon repression in Drosophila has not been identified by multi-tissue proteomics but by a finding of the Brennecke lab that the piRNA biogenesis complex has a tendency to localise to satellite DNA when the localisation to the piRNA locus is impaired. Nevertheless, the investigation of the D1 and Prod interactome is interesting and might reveal insights into the pathways that drive the formation of centromeres in a tissue specific manner.

We thank the reviewer for the overall positive comments on our manuscript. As noted above, we have performed a substantial number of revision experiments and improved our text. We believe that our

revised manuscript demonstrates a clear link between our proteomics data and the transposon repression. We would like to make three main points,

1. Our proteomics data identified that D1 and Prod co-purified transposon repression proteins in embryos. To test the functional significance of this association, we have used *Drosophila* genetics to generate flies lacking embryonic D1. In adult ovaries from these flies, we observe a striking elevation in transposon expression and Chk2-dependent gonadal atrophy. Along with the results from the control genotypes (F_1 *D1* mutant, F_2 *D1* het), our data clearly indicate that embryogenesis (and potentially early larval development) are a period when D1 establishes heritable TE silencing that can persist throughout development.
2. Based on the newly acquired RNA-seq and small RNA seq data, we have edited our title to more accurately reflect our data. Specifically, we have substituted the word ‘transgenerational’ with ‘heritable’, meaning that the presence of D1 during early development alone is sufficient to heritably repress TEs at later stages of development.
3. In addition, our RNA seq and small RNA seq experiments demonstrate that D1 makes a negligible contribution to piRNA biogenesis and TE repression in adults, despite the significant mislocalization of the RDC complex. In this regard, our results are substantially different from the recent Kipferl study from the Brennecke lab (Baumgartner et al. 2022).

Major comments

Unfortunately, the proteomic data sets are not very convincing. Not even the corresponding baits are detected in all assays. I wonder whether the extraction procedure really allows the authors to analyze all functionally relevant interactions of Prod and D1. It would be good to see a western blot or an MS analysis of the soluble nuclear extract they use for purification compared to the insoluble chromatin. It may well be that a large portion of Prod or D1 is lost in this early step.

I also find the description of the proteomic results hard to follow as the authors mostly list which proteins the find as interactors of Prod and D1 without stating in which tissue or with what bait they could purify them (i.e. p7: Importantly, our hits included known chromocenter-associated or pericentromeric heterochromatin-associated proteins, such as Su(var)3-9[52], ADD1[23,24], HP5[23,24],mei-S332[53], Mes-4[23], Hmr[24,39,54], Lhr[24,39], and members of the chromosome passenger complex, such as borr and Incenp[55]). It would be interesting to at least discuss tissue specific interactions.

Out of six total AP-MS experiments in this manuscript (D1 x 3, Prod x 2 and Piwi), we observe a strong enrichment of the bait in 5/6 attempts (log_2FC between 4-12). In the initial submission, the lack of a third high-quality biological replicate for the D1 testis sample meant that only the p-value (0.07) was not

meeting the cutoff. To address this, we have repeated this experiment with an additional biological replicate (Fig. S2A), and our data now clearly show that D1 is significantly enriched in the testis sample.

As suggested by the reviewer, we have also assessed our lysis conditions (450mM NaCl and benzonase) and the solubilization of our baits post-lysis. In Fig. S1D, we have blotted equivalent fractions of the soluble supernatant and insoluble pellet from GFP-Piwi embryos and show that both GFP-Piwi and D1 are largely solubilized following lysis. In Fig. S1E, we also show that our IP protocol works efficiently.

GFP-Prod pulldown in embryos is the only instance in which we do not detect the bait by mass spectrometry. Here, one reason could be relatively low expression of GFP-Prod in comparison to GFP-D1 (Fig. S1E), which may lead to technical difficulties in detecting peptides corresponding to Prod. However, we note that Prod IP co-purified proteins such as Bocks that were previously suggested as Prod interactors from high-throughput studies (Giot et al. 2003; Guruharsha et al. 2011). In addition, Prod IP from embryos also co-purified proteins known to associate with chromocenters such as Hcs and Saf-B. Finally, the concordance between D1 and Prod co-purified proteins from embryo lysates (Fig. 2A, C) suggest that the Prod IP from embryos is of reasonable quality.

We also acknowledge the reviewer's comment that the description of the proteomic data was hard to follow. Therefore, we have revised our text to clearly indicate which bait pulled down which protein in which tissue (lines 147-155). We have also highlighted and discussed bait-specific and tissue-specific interactions in the text (lines 157-173).

Minor comments

The authors may also want to provide a bit more information on the quantitation of the proteomic data such as how many values were derive from the match-between runs function and how many were imputed as this can severely distort the quantification.

To ensure the robustness of our data analysis, we have now only considered those proteins that were consistently identified in all replicates for the D1/Prod/Piwi pulldowns. However, it is also important to bear in mind that missing values occur when interactors are not identified in the control pulldown. In instances where the protein of interest was not identified in all control replicates, we imputed the missing values from a normal distribution generated with an average that is four times lower than the mean of the D1/Prod/Piwi sample values and a matching standard deviation. In instances where the protein of interest was identified in only one control replicate, we imputed the missing values based on the unique value identified for the control and a standard deviation matching the pulldown samples. In instances with only

one missing value fro the control replicates, we imputed using a normal distribution generated with the average and standard deviation of the control values. Detailed information regarding the imputed values is also provided (Table 1, this document). The coding script used for the data analysis is available in the PRIDE submission of the dataset (Table 2, this document). This information has been added to our methods section under ‘data availability’.

Table 1: Number of match-between-runs and imputations for embryo, ovary and testis datasets

Dataset	# match between runs	% match between runs	% imputation
Embryo_D1	5541/27543	20.11	5.81
Embryo_Prod	5541/27543	20.11	4.64
Ovary_D1	1936/9530	20.30	3.56
Ovary_Prod	1936/9530	20.30	4.34
Testis_D1	1748/7168	24.39	1.64
Piwi	5656/22542	25.09	2.48

Table 2: Access to the PRIDE submission of the datasets

Name	ID PRIDE	UN reviewer	PW reviewer
IP-MS of D1 from Testis tissue	PXD044026	reviewer_pxd044026@ebi.ac.uk	ydswDQVW
IP-MS of Piwi from Embryo tissue	PXD043237	reviewer_pxd043237@ebi.ac.uk	TMCoDsdx
IP-MS of Prod and D1 proteins from Ovary tissue	PXD043236	reviewer_pxd043236@ebi.ac.uk	VOHqPmaS
IP-MS of Prod and D1 proteins from Embryo tissue	PXD043234	reviewer_pxd043234@ebi.ac.uk	L77VXdvA

****Referee Cross-Commenting****

I agree with the two other reviewers that the connection between the interactome and the transgenerational phenotype is unclear. This is also what I meant i my comment that the title is somewhat misleading. A systematic analysis of the D1 and Prod knock down effect on mRNAs and small Rnas would indeed be helpful to better understand the interesting effect.

As suggested by the reviewer, we have performed RNA seq and small RNA seq in control and *D1* mutant ovaries (Fig. 4) to fully understand the contribution of D1 in piRNA biogenesis and TE repression. Briefly,

the mislocalization of RDC complex in D1 mutant ovaries does not significantly affect TE-mapping piRNA biogenesis (Fig. 4C, E). In addition, loss of D1 does not substantially alter TE expression in the ovaries (Fig. 4B) or alter the expression of genes involved in TE repression (Fig. 4F). Along with the results presented in Fig. 5 and Fig. 6, our data clearly indicate that embryogenesis (and potentially early larval development) is a critical period during which D1 makes an important contribution to TE repression.

Reviewer #1 (Significance (Required)):

Nevertheless, the investigation of the D1 and Prod interactome is interesting and might reveal insights into the pathways that drive the formation of centromeres in a tissue specific manner. It may be mostly interesting for the Drosophila community but could also be exiting for a broader audience interested in the connection of heterochromatin and its indirect effect on the regulation of transposable elements.

We thank the reviewer again for the helpful and constructive comments, which have enabled us to significantly improve our study. We are excited by the results from our study, which illuminate unappreciated aspects of transcriptional silencing in constitutive heterochromatin.

Reviewer #2 (Evidence, reproducibility and clarity (Required)):

Chavan et al. set out to enrich our compendium of pericentric heterochromatin-associated proteins - and to learn some new biology along the way. An ambitious AP-Mass baited with two DNA satellite-binding proteins (D1 and Prod), conducted across embryos, ovaries, and testes, yielded hundreds of candidate proteins putatively engaged at chromocenters. These proteins are enriched for a restricted number of biological pathways, including DNA repair and transposon regulation. To investigate the latter in greater depth, the authors examine D1 and prod mutants for transposon activity changes using reporter constructs for multiple elements. These reporter constructs revealed no transposon activation in the adult ovary, where many proteins identified in the mass spec experiments restrict transposons. However, the authors suggest that the D1 mutant ovaries do show disrupted localization of a key member of a transposon restriction pathway (Cuff), and infer that this mislocalization triggers a striking, transposon derepression phenotype in the F2 ovaries.

The dataset produced by the AP-Mass Spec offers chromosome biologists an unprecedented resource. The PCH is long-ignored chromosomal region that has historically received minimal attention; consequently, the pathways that regulate heterochromatin are understudied. Moreover, attempting to connect genome organization to transposon regulation is a new and fascinating area. I can easily

envision this manuscript triggering a flurry of discovery; however, there is quite a lot of work to do before the data can fully support the claims.

We appreciate the reviewer taking the time to provide thoughtful comments and constructive suggestions to improve the manuscript. We believe that we have addressed all the comments raised to the significant advantage of our paper.

Major comments

1. The introduction requires quite a radical restructure to better highlight the A) importance of the work and B) limit information whose relevance is not clear early in the manuscript.

A. Delineating who makes up heterochromatin is a long-standing problem in chromosome biology. This paper has huge potential to contribute to this field; however, it is not the first. Others are working on this problem in other systems, for example PMID:29272703. Moreover, we have some understanding of the distinct pathways that may impact heterochromatin in different tissues (e.g., piRNA biology in ovaries vs the soma). Also, the mutant phenotypes of prod and D1 are different. Fleshing out these three distinct points could help the reader understand what we know and what we don't know about heterochromatin composition and its special biology. Understanding where we are as a field will offer clear predictions about who the interactors might be that we expect to find. For example, given the dramatically different D1 and prod mutant phenotypes (and allele swap phenotypes), how might the interactors with these proteins differ? What do we know about heterochromatin formation differences in different tissues? And how might these differences impact heterochromatin composition?

The reviewer brings up a fair point and we have significantly reworked our introduction. We share the reviewer’s opinion that improved knowledge of the constitutive heterochromatin proteome will reveal novel biology.

B. The attempt to offer background on the piRNA pathway and hybrid dysgenesis in the Introduction does not work. As a naïve reader, it was not clear why I was reading about these pathways - it is only explicable once the reader gets to the final third of the Results. Moreover, the reader will not retain this information until the TE results are presented many pages later. I strongly urge the authors to shunt the two TE restriction paragraphs to later in the manuscript. They are currently a major impediment to understanding the power of the experiment - which is to identify new proteins, pathways, and ultimately, biology that are currently obscure because we have so little handle on who makes up heterochromatin.

We agree with this suggestion. We have introduced the piRNA pathway in the results section (lines 205 – 216), right before this information is needed. We've also removed the details on hybrid dysgenesis, since our new data argue against a maternal effect from the *D1* mutant.

2. The implications of the failure to rescue female fertility by the tagged versions of both D1 and Prod are not discussed. Consequently, the reader is left uneasy about how to interpret the data.

We understand this point raised by the reviewer. However, in our proteomics experiments, we have used GFP-D1 and GFP-Prod ovaries from ~1 day old females (line 579, methods). These ovaries are morphologically similar to the wild type (Fig. S1C) and their early germ cell development appears to be intact. Moreover, chromocenter formation in female GSCs is comparable to the wildtype (Fig. 1C-D). These data, along with the rescue of the viability of the Prod mutant (Fig. S1A-B), suggest that the presence of a GFP tag is not compromising D1 or Prod function in the early stages of germline development and is consistent with published and unpublished data from our lab. In addition, D1 and Prod from ovaries co-purify proteins such as Smn (D1) and CG15107 (Prod), which have been identified in previous high-throughput screens (Guruharsha et al. 2011; Tang et al. 2023). In addition, we also identify chromatin remodelers such as Mi-2 (D1) and Bap60 (Prod), which are known to function in heterochromatin formation and maintenance. For these reasons, we believe that GFP-D1 and GFP-Prod ovaries are a good starting point for the AP-MS experiment. We speculate that the failure to completely rescue female fertility may be due to improper expression levels of GFP-D1 or GFP-Prod flies at later stages of oogenesis, which are not present in ovaries from newly eclosed females and therefore unlikely to affect our proteomic data.

3. How were the significance cut-offs determined? Is the p-value reported the adjusted p-value? As a non-expert in AP-MS, I was surprised to find that the p-value, at least according to the Methods, was not adjusted based on the number of tests. This is particularly relevant given the large/unwieldy(?) number of proteins that were identified as signficant in this study. Moreover, the D1 hit in Piwi pull down is actually not significant according to their criteria of p <0.05 (D1 is p=0.05).

In our initial manuscript, we used a standard cutoff of $log_2FC>1$ and $p<0.05$ for the proteomic experiments. The p-value was indeed not adjusted based on the number of tests due to the inherently noisy nature of our AP-MS data and based on the rationale that multiple testing correction is often not considered an absolute requirement for quantitative proteomics experiments (Pascovici et al. 2016). Essentially, the cost of multiple testing corrections in quantitative proteomics (elimination of false

negatives) outweighs the benefits (elimination of false positives). However, we noted that a lot of the variability in the proteomic data (and the resultant issues in the p-values) came from missing values in the control GFP pulldowns and a noisy imputation strategy. That is, proteins whose peptides are not identified in a single replicate are automatically given an intensity value corresponding to the bottom 1% of the normal distribution, which results in large p-values. For the revised analysis, we have used more stringent data filtering and median imputation (see lines 652-665 in the materials and methods for details) and subsequently performed multiple testing corrections, such that hits are determined using a cutoff of $\log_2FC>1$ and $p_{adj}<0.1$. For the cross-bait and cross-tissues comparisons and pathway enrichment analysis (Fig. 2C, E), we have used a more relaxed cutoff of $\log_2FC>1$ and uncorrected $p<0.05$, since identical false positives across different pulldowns are very unlikely. For these analyses, we have taken the following steps to minimize the chances that we would study false positives. First, we have focused on proteins/pathways that are found in both D1 and Prod pulldowns e.g. transposon repression proteins. Second, we have prioritized our hits based on a large effect size (high $\log_2FC$ and low p-value) since these are more likely to be bona fide interactors. Third, we have used string-DB for functional analyses where we can place our hits in existing protein-protein interaction networks (Fig. S2B-D).

4. How do we know if the lack of overlap across tissues is indeed germline- or soma-specialization rather than noise?

To address this part of the comment, we have amended our text (lines 162-173) as follows,

'Interestingly, we observed a substantial overlap between D1- and Prod-associated proteins (yellow points in Fig. 2A, B, Table S1-3) from the same tissue, with 58 hits pulled down by both baits (blue arrowheads, Fig. 2C) in embryos and ovaries. This observation is consistent with the fact that both D1 and Prod occupy sub-domains within the larger constitutive heterochromatin domain in nuclei[27]. Surprisingly, only 9 proteins were co-purified by the same bait (D1 or Prod) across different tissues (magenta arrowheads, Fig. 2C, Table S1-3). In addition, only a few proteins such as an uncharacterized DnaJ-like chaperone, CG5504, were associated with both D1 and Prod in embryos and ovaries (Fig. 2D). One interpretation of these results is that the protein composition of chromocenters may be tailored to cell- and tissue-specific functions in *Drosophila*. However, more targeted experiments are required to validate whether certain proteins are indeed tissue-specific interactors of D1 and Prod.'

To make this inference, conducting some validation would be required. More generally, I was surprised to see no single interactor validated by reciprocal IP-Westerns to validate the Mass-Spec results, though I

am admittedly only adjacent to this technique. Note that colocalization, to my mind, does not validate the AP-MS data - in fact, we would a priori predict that piRNA pathway members would co-localize with PCH given the enrichment of piRNA clusters there.

Here, we would point out that we have conducted multiple validation experiments with a specific focus on the functional significance of the associations between D1/Prod and TE repression proteins in embryos. While the reviewer suggests that piRNA pathway proteins may be expected to enrich at the pericentromeric heterochromatin, this is not always the case. For example, Piwi and Mael are present across the nucleus (pulled down by D1/Prod in embryos) while Cutoff, which is present adjacent to chromocenters in nurse cells, was not observed to interact with either D1 or Prod in the ovary samples.

Therefore, for Piwi, we performed a MS experiment in embryos due to the higher sensitivity of this method (GFP-Piwi AP-MS, Fig. 3D). Excitingly, this experiment revealed that four largely uncharacterized proteins (CG14715, CG10208, Ugt35D1 and Fit) were highly enriched in the D1, Prod and Piwi pulldowns and these proteins will be an interesting cohort for future studies on transposon repression in *Drosophila* (Fig. 3C, D).

Furthermore, we believe that determining the localization of proteins co-purified by D1/Prod is an important and orthogonal validation approach. For Sov, which is implicated in piRNA-dependent heterochromatin formation, we observe foci that are in close proximity to D1- and Prod-containing chromocenters (Fig. 3B).

As for suggestion to validate by IP-WBs, we would point out that chromocenters exhibit properties associated with phase separated biomolecular condensates. Based on the literature, these condensates tend to associate with other proteins/condensates through low affinity or transient interactions that are rarely preserved in IP-WBs, even if there are strong functional relationships. One example is the association between D1 and Prod, which do not pull each other down in an IP-WB (Jagannathan et al. 2019), even though D1 and Prod foci dynamically associate in the nucleus and mutually regulate each other’s ability to cluster satellite DNA repeats (Jagannathan et al. 2019). Similarly, IP-WB using GFP-Piwi embryos did not reveal an interaction with D1 (Fig. S4B). However, our extensive functional validations (Figures 4-6) have revealed a critical role for D1 in heritable TE repression.

5. The AlphaFold2 data are very interesting but seem to lack of negative control. Is it possible to incorporate a dataset of proteins that are not predicted to interact physically to elevate the impact of the

ones that you have focused on? Moreover, the structural modeling might suggest a competitive interaction between D1 and piRNAs for Piwi. Is this true? And even if not, how does the structural model contribute to your understanding for how D1 engages with the piRNA pathway? The Cuff mislocalization?

In the revised manuscript, we have generated more structural models using AlphaFold Multimer (AFM) for proteins ($log_2FC>2$, $p<0.01$) co-purified by D1/Prod from all tissues (Table S4) and for all protein pairs from CG14715, CG10208, Ugt35D1, Fit, D1, Prod and Piwi (Fig. 3E, Table S8). In our opinion, the AFM models are not meant to validate our proteomic data (which have their own negative controls and functional validations). Rather, the purpose of using AFM was to differentiate between potential direct and indirect interactions by determining medium- and high-scoring interaction interfaces (ipTM >0.5 and ipTM>0.8), now elaborated in lines 175-182. Despite the extensive disorder in D1 and Prod, we identified 22 proteins, including Piwi, that yield interfaces with ipTM scores >0.5 (Table S4, Table S8). These hits are promising candidates to further understand D1 and Prod function in the future.

For the predicted model between Prod/D1 and Piwi (Fig. S4A), one conclusion could indeed be competition between D1/Prod and piRNAs for Piwi. Another possibility is that a transient interaction mediated by disordered regions on D1/Prod could recruit Piwi to satellite DNA-embedded TE loci in the pericentromeric heterochromatin. These types of interactions may be especially important in the early embryonic cycles, where repressive histone modifications such as H3K9me2/3 must be deposited at the correct loci for the first time. We suggest that mutating the disordered regions on D1 and Prod to potentially abrogate the interaction with Piwi will be important for future studies.

6. The absence of a TE signal in D1 and Prod mutant ovaries would be much more compelling if investigated more agnostically. The observation that not all TE reporter constructs show a striking signal in the F2 embryos makes me wonder if Burdock and gypsy are not regulated by these two proteins but possibly other TEs are. Alternatively, small RNA-seq would more directly address the question of whether D1 and Prod regulate TEs through the piRNA pathway.

We completely agree with this comment from the reviewer. We have performed RNA seq on *D1* heterozygous (control) and *D1* mutant ovaries in a *chk2*^{6006} background. Since Chk2 arrests germ cell development in response to TE de-repression and DNA damage(Ghabrial and Schüpbach 1999; Moon et al. 2018), we reasoned that the *chk2* mutant background would prevent developmental arrest of potential TE-expressing germ cells and we observed that both genotypes exhibited similar gonad morphology (Fig. 4A). From our analysis, we do not observe a significant effect on TE expression in the absence of D1,

except for the LTR retrotransposon *tirant* (Fig. 4B). We also do not observe differential expression of TE repression genes (Fig. 4F).

We have complemented our RNA seq experiment with small RNA profiling from *D1* heterozygous (control) and *D1* mutant ovaries. Here, overall piRNA production and antisense piRNAs mapping to TEs were largely unperturbed (Fig. 4C-E).

Overall, our data is consistent with the TE reporter data (Fig. S7) and suggests that zygotic depletion of D1 does not have a prominent role in TE repression. However, we have uncovered that the presence of D1 during embryogenesis is critical for TE repression in adult gonads (Fig. 6, Fig. S9).

7. I had trouble understanding the significance of the Cuff mis-localization when D1 is depleted. Given Cuff's role in the piRNA pathway and close association with chromatin, what would the null hypothesis be for Cuff localization when a chromocenter is disrupted? What is the null expectation of % Cuff at chromocenter given that the chromocenter itself expands massively in size (Figure 4D). The relationship between these two factors seems rather indirect and indeed, the absence of Cuff in the AP would suggest this. The biggest surprise is the absence of TE phenotype in the ovary, given the Cuff mutant phenotype - but we can't rule out given the absence of a genome-wide analysis. I think that these data leave the reader unconvinced that the F2 phenotype is causally linked to Cuff mislocalization.

We apologize that this data was not more clearly represented. In a wild-type context, Cuff is distributed in a punctate manner across the nurse cell nuclei, with the puncta likely representing piRNA clusters (Fig. 5A-B). We find that a small fraction of Cuff (~5%) is present adjacent to the nurse cell chromocenter (inset, Fig. 5A and Fig. 5D). In the absence of D1, the nurse cell chromocenters increase ~3-4 fold in size. However, the null expectation is still that ~5% of total Cuff would be adjacent to the chromocenter, since the piRNA clusters are not expected to expand in size. In reality, we observe ~27% of total Cuff is mislocalized to chromocenters. Our data indicate that the satellite DNA repeats at the larger chromocenters must be more accessible to Cuff in the *D1* mutant nurse cells. This observation is corroborated by the significant increase in piRNAs corresponding to the 1.688 satellite DNA repeat family (Fig. 4E).

The lack of TE expression in the F_1 *D1* mutant was indeed surprising based on the Cuff mislocalization but as the reviewers pointed out, we only analyzed two TE reporter constructs in the initial submission. In the revised manuscript, we have performed both RNA seq and small RNA seq. Surprisingly, our data reveal that the Cuff mislocalization does not significantly affect piRNA biogenesis (Fig. 4C, D) and piRNAs

mapping to TEs. As a result, both TE repression (Fig. 4B) and fertility (Fig. 6D) are largely preserved in the absence of D1 in adult ovaries.

Finally, we thank the reviewer for their excellent suggestion to incorporate the F_2 *D1* heterozygote (Fig. S9) in our analysis! This important control has revealed that the maternal effect of the *D1* mutant is negligible for gonad development and fertility (Fig. 6B-D). Rather, our data clearly emphasize embryogenesis (or early larval development) as a key period during which D1 can promote heritable TE repression. Essentially, D1 is not required during adulthood for TE repression if it was present in the early stages of development.

8. Apologies if I missed this, but Figure 5 shows the F2 D1 mutant ovaries only. Did you look at the TM6 ovaries as well? These ovaries should lack the maternally provisioned D1 (assuming that females are on the right side) but have the zygotic transcription.

As mentioned above, this was a great suggestion and we've now characterized this important control in the context of germline development and fertility, to the significant advantage of our paper.

Minor comments

9. Add line numbers for ease of reference

We apologize for this. Line numbers have been added in the full revision.

10. The function of satellite DNA itself is still quite controversial - I would recommend being a bit more careful here - the authors could refer instead to genomic regions enriched for satellite DNA are linked to xyz function (see Abstract line 2 and 7, for example.)

The abstract has been rewritten and does not directly implicate satellite DNA in a specific cellular function. However, we have taken the reviewer's suggestion in the introduction (line 57-58).

11. "Genetic conflicts" in the introduction needs more explanation.

This sentence has been removed from the introduction in the revised manuscript.

12. "In contrast" is not quite the right word. Maybe "However" instead (1st line second paragraph of Intro)

Done. Line 57 of the revised manuscript.

13. Results: what is the motivation for using GSC-enriched testis?

We use GSC-enriched testes for practical reasons. First, they contain a relatively uniform population of mitotically dividing germ cells unlike regular testes which contain a multitude of post-mitotic germ cells such as spermatocytes, spermatids and sperm. Second, GSC-enriched testes are much larger than normal testes and reduced the number of dissections that were needed for each replicate.

14. Clarify sentence about the 500 proteins in the Results section - it's not clear from context that this is the union of all experiments.

Done. Lines 145-149 in the revised manuscript.

15. The data reported are not the first to suggest that satellite DNA may have special DNA repair requirements. e.g., PMID: 25340780

We apologize if we gave the impression that we were making a novel claim. Specialized DNA repair requirements at repetitive sequences have indeed been previously hypothesized(Charlesworth et al. 1994) and studied and we have altered the text to better reflect this (lines 193-195). We have cited the study suggested by the reviewer as well as studies from the Chiolo(Chiolo et al. 2011; Ryu et al. 2015; Caridi et al. 2018) and Soutoglou(Mitrentsi et al. 2022) labs, which have also addressed this fascinating question.

16. Page 10: indicate-> indicates.

Done.

17. Page 14: revise for clarity: "investigate a context whether these interactions could not take place"

We've incorporated this suggestion in the revised text (lines 383-386).

18. Might be important to highlight the 500 interactions are both direct and indirect. "Interacting proteins" alone suggests direct interactions only.

Done. Lines 145-149.

19. The effect of the aub mutant on chromocenter foci did not seem modest to me - however, the bar graphs obscure the raw data - consider plotting all the data not just the mean and error?

Done. This data is now represented by a box-and-whisker plot (Fig. S5), which shows the distribution of the data.

Reviewer #2 (Significance (Required)):

The dataset produced by the AP-Mass Spec offers chromosome biologists an unprecedented resource. The PCH is long-ignored chromosomal region that has historically received minimal attention; consequently, the pathways that regulate heterochromatin are understudied. Moreover, attempting to connect genome organization to transposon regulation is a new and fascinating area. I can easily envision this manuscript triggering a flurry of discovery; however, there is quite a lot of work to do before the data can fully support the claims.

This manuscript represents a significant contribution to the field of chromosome biology.

We thank the reviewer for the positive evaluation of our manuscript, and we really appreciate the great suggestion for the F_2 D1 heterozygote control! Overall, we believe that our manuscript is substantially improved with the addition of RNA seq, small RNA seq and important genetic controls. Moreover, we are excited by the potential of our study to open new doors in the study of pericentromeric heterochromatin.

Reviewer #3 (Evidence, reproducibility and clarity (Required)):

In the manuscript entitled "Multi-tissue proteomics identifies a link between satellite DNA organization and transgenerational transposon repression", the authors used two satellite DNA-binding proteins, D1 and Prod, as baits to identify chromocenter-associated proteins through quantitative mass spectrometry. The proteomic analysis identified ~500 proteins across embryos, ovaries, and testes, including several piRNA pathways proteins. Depletion of D1 or Prod did not directly contribute to transposon repression in ovary. However, in the absence of maternal and zygotic D1, there was a dramatic increase of agametic ovaries and transgenerational transposon de-repression. Although the study provides a wealth of proteomic data, it lacks mechanistic insights into how satellite DNA organization influence the interactions with other proteins and their functional consequences.

We thank the reviewer for highlighting that this study will be a valuable resource for future studies on the composition and function of pericentromeric heterochromatin. Based on the reviewer's request for more mechanistic knowledge into how satellite DNA organization affects transposon repression, we have performed RNA seq and small RNA seq, added important genetic controls and significantly improved our text. In the revised manuscript, our data clearly demonstrate that embryogenesis (and potentially early larval development) is a critical time period when D1 contributes to heritable TE repression. Flies lacking D1 during embryogenesis exhibit TE expression in germ cells as adults, which is associated with Chk2-dependent gonadal atrophy. We are particularly excited by these data since TE loci are embedded in the satellite DNA-rich pericentromeric heterochromatin and our study demonstrates an important role for a satellite DNA-binding protein in TE repression.

Major comments

1. While the identification of numerous interactions is significant, it would be helpful to acknowledge that lots of these proteins were known to bind DNA or heterochromatin regions. To strengthen the study, I recommend conducting functional validation of the identified interactions, in addition to the predictions made by Alphfold 2.

We are happy to take this comment on board. In fact, we believe that the large number of DNA-binding and heterochromatin-associated proteins identified in this study are a sign of quality for the proteomic datasets. In the revised manuscript, we have highlighted known heterochromatin proteins co-purified by D1/Prod as well as proteins previously suggested to interact with D1/Prod from high-throughput studies in lines 148-155 (Guruharsha et al. 2011; Tang et al. 2023). In this study, we have focused on the previously unknown associations between D1/Prod and TE repression proteins and functionally validated these interactions as presented in Figures 3-6.

2. The observation of transgenerational de-repression is intriguing. However, to better support this finding, it would be better to provide a mechanistic explanation based on the data presented.

We appreciate this comment from the reviewer, which is similar to major comment #6 raised by reviewer #2. To generate mechanistic insight into the underlying cause of gonad atrophy in the F_2 D1 mutant, we have performed RNA seq, small RNA seq and analyzed germline development and fertility in the F_2 D1 heterozygous control (Fig. S9).

For the RNA seq, we used *D1* heterozygous (control) and *D1* mutant ovaries in a *chk2*^{6006} background. Since Chk2 arrests germ cell development in response to TE de-repression and DNA damage(Ghabrial

and Schüpbach 1999; Moon et al. 2018), we reasoned that the *chk2* mutant background would prevent developmental arrest of potential TE-expressing germ cells and we observed that both genotypes exhibited similar gonad morphology (Fig. 4A). From our analysis, we do not observe a significant effect on TE expression in the absence of D1, except for the LTR retrotransposon *tirant* (Fig. 4B). We also do not observe differential expression of TE repression genes (Fig. 4F).

We have complemented our RNA seq experiment with small RNA profiling from *D1* heterozygous (control) and *D1* mutant ovaries. Here, overall piRNA production and antisense piRNAs mapping to TEs were largely unperturbed (Fig. 4C-E). Together, these data are consistent with the TE reporter data (Fig. S7) and suggests that zygotic depletion of D1 does not have a prominent role in TE repression.

However, we have uncovered that the presence of D1 during embryogenesis is critical for TE repression in adult gonads (Fig. 6, Fig. S9). Essentially, either only maternal deposited D1 (F_1 *D1* mutant) or only zygotically expressed D1 (F_2 *D1* het) were sufficient to ensure TE repression and fertility. In contrast, a lack of D1 during embryogenesis (F2 D1 mutant) led to elevated TE expression and Chk2-mediated gonadal atrophy.

Our results also explain why previous studies have not implicated either D1 or Prod in TE repression, since D1 likely persists during embryogenesis when using depletion approaches such as RNAi-mediated knockdown or F_1 generation mutants.

Minor comments

3. Given the maternal effect of the D1 mutant, in Figure 4, I suggest analyzing not only nurse cells but also oocytes to gain a more comprehensive understanding.

We agree with the reviewer that this experiment can be informative. In the F_2 *D1* mutant ovaries, germ cell development does not proceed to a stage where oocytes are specified, thus limiting microscopy-based approaches. Nevertheless, we have gauged oocyte quality in all the genotypes using a fertility assay (Fig. 6D) since even ovaries that have a wild-type appearance can produce dysfunctional gametes. In this experiment, we observe that fertility is largely intact in the F_1 *D1* mutant and F_2 *D1* heterozygote strains, suggesting that it is the presence of D1 during embryogenesis (or early larval development) that is critical for heritable TE repression and proper oocyte development.

4. The conclusion that D1 and Prod do not directly contribute to the repression of transposons needs

further analysis from RNA-seq data. Alternatively, the wording could be adjusted to indicate that D1 and Prod are not required for specific transposon silencing, such as Burdock and gypsy.

Agreed. We have performed RNA-seq in *D1* heterozygous (control) and *D1* mutant ovaries in a $chk2^{6006}$ background (Fig. 4A, B) as described above.

5. As D1 mutation affects Cuff nuclear localization, it would be insightful to sequence the piRNA in the ovaries.

Agreed. We have performed small RNA profiling from *D1* heterozygous (control) and *D1* mutant ovaries. Despite the significant mislocalization of the RDC complex, overall piRNA production and antisense piRNAs mapping to TEs were largely unaffected (Fig. 4C-E). However, we observed significant changes in piRNAs mapping to complex satellite DNA repeats (Fig. 4D), but these changes were not associated with a maternal effect on germline development or fertility (F_2 *D1* heterozygote, Fig. 6B-D).

Referee Cross-Commenting

I appreciate the valuable insights provided by the other two reviewers regarding this manuscript. I concur with their assessment that substantial improvements are needed before considering this manuscript for publication.

1. The proteomics data has the potential to be a valuable resource for other scientific community. However, I share the concerns raised by reviewer 1 about the current quality of the data sets. Addressing this, it will augment the manuscript with additional data to show the success of the precipitation. Moreover, as reviewer 2 and I suggested, additional co-IP validations of the IP-MS results are needed to enhance the reliability.

In the revised manuscript, we have performed multiple experiments to address the quality of the MS datasets based on comments raised by reviewer #1. They are as follows,

Out of six total AP-MS experiments in this manuscript (D1 x 3, Prod x 2 and Piwi), we observe a strong enrichment of the bait in 5/6 attempts (log_2FC between 4-12, Fig. 2A, B, Fig. S2A, Table S1-S3, Table S7). In the D1 testis sample from the initial submission, the lack of a third biological replicate meant that only the p-value (0.07) was not meeting the cutoff. To address this, we have repeated this experiment

with an additional biological replicate (Fig. S2A), and our data now clearly show that D1 is also significantly enriched in the testis sample.

As suggested by the reviewer #1, we have assessed our lysis conditions (450mM NaCl and benzonase) and the solubilization of our baits post-lysis. In Fig. S1D, we have blotted equivalent fractions of the soluble supernatant and insoluble pellet from GFP-Piwi embryos and show that both GFP-Piwi and D1 are largely solubilized following lysis. In Fig. S1E, we also show that our IP protocol works efficiently.

The only instance in which we do not detect the bait by mass spectrometry is for GFP-Prod pulldown in embryos. Here, one reason could be relatively low expression of GFP-Prod in comparison to GFP-D1 (Fig. S1E), which may lead to technical difficulties in detecting peptides corresponding to Prod. However, we note that Prod IP from embryos co-purified proteins such as Bocks that were previously suggested as Prod interactors from high-throughput studies (Giot et al. 2003; Guruharsha et al. 2011). In addition, Prod IP from embryos also co-purified proteins known to associate with chromocenters such as Hcs(Reyes-Carmona et al. 2011) and Saf-B(Huo et al. 2020). Finally, the concordance between D1 and Prod co-purified proteins from embryo lysates (Fig. 2A, C) suggest that the Prod IP from embryos is of reasonable quality.

As for the IP-WB validations, we would point out that chromocenters exhibit properties associated with phase separated biomolecular condensates. In our experience, these condensates tend to associate with other proteins/condensates through low affinity or transient interactions that are rarely preserved in IP-WBs, even if there are strong functional relationships. One example is the association between D1 and Prod, which do not pull each other down in an IP-WB (Jagannathan et al. 2019), even though D1 and Prod foci dynamically associate in the nucleus and mutually regulate each other's ability to cluster satellite DNA repeats (Jagannathan et al. 2019). Similarly, IP-WB using GFP-Piwi embryos did not reveal an interaction with D1 (Fig. S4B). However, our extensive functional validations (Figures 4-6) have revealed a critical role for D1 in heritable TE repression.

2. I agree with reviewer 2 that the present conclusion is not appropriate regarding D1 and Prod do not contribute to transposon silencing. As reviewer 2 and I suggested, the systematical analysis by using both mRNA-seq and small RNA-seq is required to draw the conclusion.

Agreed. We have performed RNA seq and small RNA seq as elaborated above. Our conclusions on the role of D1 in TE repression are now much stronger.

3. The transgenerational phenotype is an intriguing aspect of the study. I agree with reviewer 2 that the inclusion of TM6 ovaries would be a nice control. Further, it is hard to connect this phenotype with the interactions identified in this manuscript. It would be appreciated if the author could provide a mechanistic explanation.

We have significantly improved these aspects of our study in the revised manuscript. Through the analysis of germline development in the F_2 *D1* heterozygotes as suggested by reviewer #2, in addition to the recommended RNA seq and small RNA seq, we have now identified embryogenesis (and potentially early larval development) as a time period when D1 makes an important contribution to TE repression. Loss of D1 during embryogenesis leads to TE expression in adult germline cells, which is associated with Chk2-dependent gonadal atrophy. Taken together, we have pinpointed the specific contribution of a satellite DNA-binding protein to transposon repression.

Reviewer #3 (Significance (Required)):

Although this study successfully identified several interactions, the authors did not fully elucidate how these interactions contribute to the transgenerational silencing of transposons or ovary development.

We thank the reviewer for the thoughtful comments and overall positive evaluation of our study, especially the proteomic dataset. We are confident that the revised manuscript has improved our mechanistic understanding of the contribution made by a satellite DNA-binding protein in TE repression.

References

Baumgartner L, Handler D, Platzer SW, Yu C, Duchek P, Brennecke J. 2022. The Drosophila ZAD zinc finger protein Kipferl guides Rhino to piRNA clusters eds. D. Bourc'his, K. Struhl, and Z. Zhang. *eLife* **11**: e80067.

Caridi CP, D'Agostino C, Ryu T, Zapotoczny G, Delabaere L, Li X, Khodaverdian VY, Amaral N, Lin E, Rau AR, et al. 2018. Nuclear F-actin and myosins drive relocalization of heterochromatic breaks. *Nature* **559**: 54–60.

Charlesworth B, Sniegowski P, Stephan W. 1994. The evolutionary dynamics of repetitive DNA in eukaryotes. *Nature* **371**: 215–220.

Chiolo I, Minoda A, Colmenares SU, Polyzos A, Costes SV, Karpen GH. 2011. Double-strand breaks in heterochromatin move outside of a dynamic HP1a domain to complete recombinational repair. *Cell* **144**: 732–744.

Ghabrial A, Schüpbach T. 1999. Activation of a meiotic checkpoint regulates translation of Gurken during Drosophila oogenesis. *Nat Cell Biol* **1**: 354–357.

Giot L, Bader JS, Brouwer C, Chaudhuri A, Kuang B, Li Y, Hao YL, Ooi CE, Godwin B, Vitols E, et al. 2003. A protein interaction map of Drosophila melanogaster. *Science* **302**: 1727–1736.

Guruharsha KG, Rual JF, Zhai B, Mintseris J, Vaidya P, Vaidya N, Beekman C, Wong C, Rhee DY, Cenaj O, et al. 2011. A protein complex network of Drosophila melanogaster. *Cell* **147**: 690–703.

Huo X, Ji L, Zhang Y, Lv P, Cao X, Wang Q, Yan Z, Dong S, Du D, Zhang F, et al. 2020. The Nuclear Matrix Protein SAFB Cooperates with Major Satellite RNAs to Stabilize Heterochromatin Architecture Partially through Phase Separation. *Molecular Cell* **77**: 368-383.e7.

Jagannathan M, Cummings R, Yamashita YM. 2019. The modular mechanism of chromocenter formation in Drosophila eds. K. VijayRaghavan and S.A. Gerbi. *eLife* **8**: e43938.

Mitrentsi I, Lou J, Kerjouan A, Verigos J, Reina-San-Martin B, Hinde E, Soutoglou E. 2022. Heterochromatic repeat clustering imposes a physical barrier on homologous recombination to prevent chromosomal translocations. *Molecular Cell* **82**: 2132-2147.e6.

Moon S, Cassani M, Lin YA, Wang L, Dou K, Zhang ZZ. 2018. A Robust Transposon-Endogenizing Response from Germline Stem Cells. *Dev Cell* **47**: 660-671 e3.

Pascovici D, Handler DCL, Wu JX, Haynes PA. 2016. Multiple testing corrections in quantitative proteomics: A useful but blunt tool. *PROTEOMICS* **16**: 2448–2453.

Reyes-Carmona S, Valadéz-Graham V, Aguilar-Fuentes J, Zurita M, León-Del-Río A. 2011. Trafficking and chromatin dynamics of holocarboxylase synthetase during development of Drosophila melanogaster. *Molecular Genetics and Metabolism* **103**: 240–248.

Ryu T, Spatola B, Delabaere L, Bowlin K, Hopp H, Kunitake R, Karpen GH, Chiolo I. 2015. Heterochromatic breaks move to the nuclear periphery to continue recombinational repair. *Nat Cell Biol* **17**: 1401–1411.

Full Revision

Tang H-W, Spirohn K, Hu Y, Hao T, Kovács IA, Gao Y, Binari R, Yang-Zhou D, Wan KH, Bader JS, et al. 2023. Next-generation large-scale binary protein interaction network for Drosophila melanogaster. *Nat Commun* **14**: 2162.
