# Supplementary material for: Multi-tissue characterization of the constitutive heterochromatin proteome in Drosophila identifies a link between satellite DNA organization and transposon repression
Source: PLoS Biol. 2025 Jan 15;23(1):e3002984. doi: 10.1371/journal.pbio.3002984 (PMC11734925; doi:10.1371/journal.pbio.3002984)
Supplement: Attachment — Submitted filename: Response to Reviewers.docx [file pbio.3002984.s029.docx]

**Reviewer #1:** Comments to Chavan et al.
The authors addressed all my comments in a satisfying manner. The proteomic data are now much better described and the new RNA-Seq data clearly distinguish the effects of D1 from the ones of the RDC complex. The authors also made several editorial changes making the manuscript much easier to read.

We thank the reviewer for their constructive feedback over the course of the revisions and the positive evaluation of the revised manuscript.

**Reviewer #2:** In this revised manuscript, Chavan et al. have substantially improved the clarity, rigor, and depth of their report by revisions to the text and the addition of many new experiments, including additional QC on the proteomics experiments, RNA-seq/piRNA-seq experiments, and a deeper analysis of the D1 mutants. 

In short, the proteomics data offer a unique resource to the community while the more focused analysis linking the piRNA pathway and D1 illuminates new players in small RNA biogenesis and TE repression in an already well-studied system. Please see below for my major and minor comments on this new version of the manuscript:

We appreciate the positive comments from the reviewer, whose input has helped improve our manuscript over the course of the revisions. Please see point-by-point responses below.

**Major comments:**
1.	I was surprised to learn that the single IP-WB could not validate the Piwi:D1 interaction. The explanation of the interaction occurring in only a few cells makes sense given that the authors had to wait until the F2 generation to detect a defect; however, why would such a rare interaction in the larger embryo (or a transient one, as was also suggested) be detect robustly by the AP-MS? If this is simply a detection issue, the authors should assert that for the uninitiated.

Indeed, we believe that this discrepancy arises due to the much higher sensitivity of mass spectrometry. We have amended the text as follows in lines 257-262, ‘At the same time, we were unable to detect a Piwi-D1 interaction by co-immunoprecipitation from embryonic lysates (Fig. S4B), suggesting that this interaction may only occur in a small subset of cells (e.g. germ cells) in embryos or that the interaction may be mediated by weak/transient associations between disordered regions, *with* *both possibilities necessitating higher sensitivity approaches like mass spectrometry*.’

2.	I'm afraid the original TE data, the figures of which have been shunted to the supplement, do not add to the main text. I'm aware that this was a ton of work but it's distracting now that the authors have supplied RNA-seq data.

We appreciate the perspective of the reviewer. However, we’d point out that the experimental designs for the RNA-seq and the reporters are not completely identical (a reporter background vs a *chk2* mutant background). In addition, the reporter experiments also allow us to test the role of Prod in ovary TE expression, which is not otherwise assessed in the RNA-seq experiment. Finally, we consider that the concordance between the RNA-seq and the reporter data increases confidence in our conclusions that TE expression is not especially affected in the ovaries of the F_1_ *D1* mutant. Therefore, we would like to keep Fig. S6 and Fig. S7 in the manuscript.

3.	The authors mention that tirant was a rare TE upregulated in the D1 mutant ovaries; however, the piRNA data suggest that this upregulation is not the production of piRNA pathway perturbations. This inconsistency should be addressed. Is tirant upregulation independent of the piRNA pathway? Related: the authors might consider inverting the presentation to show the piRNA-seq first and RNA-seq second given the a priori hypothesis that that the piRNA pathway should be disrupted and TE repression would the product of that.

The recent invasion of *tirant* (Schwarz et al. 2021)means that its copy number can be highly variable across standard wildtype strains, including those likely used to generate many of the mutant strains used in our study. We have now indicated as such in lines 316-317 as follows,

‘One exception was the LTR retrotransposon *tirant,* which has recently invaded *D. melanogaster* populations(Schwarz et al. 2021), and was expressed 5-6-fold higher in *D1* mutant ovaries.’

In addition, *tirant* expression has been shown to occur independently of piRNA-based regulation(Wang et al. 2020; Parhad et al. 2017), which is similar to our findings. However, why this is the case remains unclear. In the manuscript, we have cited those previous studies and amended the text in lines 335-336 as follows,

‘Rather, we propose that impaired heterochromatin stability or other piRNA-independent factors in the *D1* mutant could be the underlying cause’

4.	The most dramatic phenotype is the ovary atrophy in the F2 ovaries. However, these were not subjected to RNA-seq and instead a (very convincing) copia RNA FISH experiment. There was no clear justification for 1. Focusing on copia (is copia a particularly excellent TE for learning about a larger disruption to TE silencing?). And 2. Why assaying copia piRNAs is not necessary for implicating the piRNA pathway. If these cannot be addressed in the text, an RNA-seq experiment (and possibly a piRNA-seq experiment) should be performed.

The agametic ovaries in the F_2_ *D1* mutant are not particularly suitable for RNAseq and small RNAseq experiments, due to the paucity of germ cells. Therefore, we turned to RNA FISH to determine if certain TEs were expressed in the remnant few germ cells of these agametic ovaries. The choice of TEs was mainly based on available reagents and we tested *copia*, *burdock* and *gypsy* and *Het-A* (indicated in line 418-420). Of these, we noted a strong expression of *copia*, but not the other TEs, in the F_2_ *D1* mutant ovaries. It is completely plasusible that other TEs are also expressed in the F_2_ *D1* mutant ovaries. It is also possible that the TE expression may be driven by decreased piRNA biogenesis or other defects associated with heterochromatin establishment in the absence of D1. However, in our opinion, these experiments are a separate project and fall outside the scope of this manuscript.

To incorporate the suggestions of the reviewer, we have amended the text as follows,

In the results (line 426-428): ‘Thus, *the high expression of copia, and potentially other TEs*, occurring in the early germ cells of F_2_ *D1* mutants, may contribute to the loss of GSCs or block their differentiation, ultimately leading to agametic gonads’

In the discussion (Line 465-470): *‘We consider it likely that the TE expression in the F_2_ D1 mutant germ cells results from heterochromatin instability. One straightforward possibility is that loss of heterochromatin at TE loci directly enables transposon expression. However, since piRNA biogenesis relies on heterochromatin modifications such as H3K9me3 and H3K27me3(Rangan et al. 2011; Akkouche et al. 2024), it is also possible that impaired piRNA biogenesis in the F_2_ D1 mutant germ cells may contribute to TE expression and gonadal atrophy.’*

**Minor comments:**
5.	More cautious language should be used around the link between TE depression and Chk2 rescue. The authors detect many DNA repair proteins interacting directly or indirectly with D1. The disruption to DNA repair at satellite-rich DNA could account for the DNA damage as well, no?

Agreed. We have added the following text to the discussion (lines 470-475), ‘Interestingly, germ cell loss in the F_2_ *D1* mutant ovaries was nearly completely rescued by *chk2* mutation. While *chk2*-dependent GSC loss can be triggered by mutations in the piRNA pathway, *chk2* also responds to unresolved DNA double strand breaks (DSBs) in Drosophila female GSCs(Ma et al. 2016). Since D1 interacts with a many DNA repair proteins, it is possible that persistent DSBs in F_2_ *D1* mutant germ cells, in addition to TE expression, may contribute to *chk2*-dependent germ cell loss.’

6.	Abstract Line 40/41: transcriptomics and small RNA profiling was not used for the F2 phenotypic analysis - only RNA FISH was, correct?

Strictly speaking, yes, and we understand the point being raised. However, in the context of the study, the genetics, transcriptomics and small RNA profiling were all essential to attribute the observed TE expression to presence of D1 during embryogenesis. Therefore, in our opinion, this line is not misleading and we have left it as is.

7.	Figure 2A: Are there no proteins that both repress TEs and were common to the two experiments, as the color coding suggests (there is no category for both)?

There are a few genes in common in the DNA repair and transposon repression lists, which can be easily found in Supplementary Table 5 and Supplementary Table 6. For example, D1 in embryos co-purified Caf1-180 and Tfb1, both of which are annotated as DNA repair and transposon repression proteins. Since there are three categories in Fig. 2A, B (DNA repair, transposon repression and D1/Prod common interactors), we chose not to overcomplicate the figure but rather prioritized the categorization as follows, transposon repression > DNA repair > D1/Prod common interactors. This info has been added to the figure legend in lines 1090-1092, ‘For proteins belonging to more than one category, we prioritized the representation as follows, transposon repression > DNA repair > D1/Prod common interactors.’

8.	Lines 147-155: Observing these positive controls in the dataset is certainly satisfying. However, the authors failed to mention any known interactors that did not show up in their new dataset.

D1 and Prod are not particularly well studied in terms of their protein-protein interactions and there are no ‘gold standard’ interactions that we expected to observe but did not see in our MS dataset. Rather than list all of the putative D1/Prod interactions or heterochromatin proteins we did not co-purify, we have changed the text (line 153-156) as follows, ‘Our proteomic data also identified some of the previously reported D1/Prod associations such as D1-Xpc (embryo), D1-Stwl (embryo), D1-Smn (ovary), D1-CG5599 (embryo), D1-CG42232 (embryo), Prod-CG15107 (ovary) and Prod-bocks (embryo)(Guruharsha et al. 2011; Tang et al. 2023).’

9.	Fig 2C: Is LogFC >1 an agreed upon cut-off for this type of data? A LogFC of 1.1 is not especially compelling to the outside reader. Similar, in Fig 2D: why are proteins displayed that have a p[adj] >0.1?

We believe that the Log_2_FC >1 cutoff is reasonable, especially when the adjusted p-values are also taken into account. For Fig. 2D, we have removed the proteins that didn’t satisfy the p[adj] < 0.1cutoff.

10.	Fig 3A: showing "co-localization" with a protein that diffusely marks the whole nucleus seems rather uninformative; moreover, the absence of single channels would make it difficult to see overlap if indeed it was informative.

In the revised manuscript, we merely state in lines 227-230 that ‘Although Piwi mediates transcriptional repression at TE loci, it is localized throughout the entire nucleus and the GFP-Piwi signal expectedly overlapped with the heterochromatic D1 and Prod foci in embryonic nuclei (Fig. 3A)’. Due to the presence of Piwi across the nucleus, we intentionally do not make any statements about co-localization.

11.	Fig 5: more striking than the elevated 359bp signal is probably the shift from distinct foci to a single, large blob-like signal. Given that D1 is a sort of architectural protein, this interesting finding requires at least some mention.

The ‘blob-like’ 359bp satellite RNA signal is almost certainly due to the Cuff mislocalization to the expanded chromocenters in the *D1* mutant, which is already emphasized in lines 368-380. The presence of the single blob in the *D1* mutant nurse cell in Fig. 5F could indeed be due to reorganization of the 359bp satellite DNA loci in the nucleus, or merely due to the confocal slice presented. While potentially interesting, we feel that this observation needs more investigation.

12.	The RNAaseH experiment lacks a positive control without the treatment.
The ovaries lackig the RNaseH treatment are presented in Fig. 5F. The samples in Fig. 5F and Fig. 5H were treated exactly the same, with the exception of RNase H treatment.

**References**

Akkouche A, Kneuss E, Bornelöv S, Renaud Y, Eastwood EL, Lopik J van, Gueguen N, Jiang M, Creixell P, Maupetit-Mehouas S, et al. 2024. A dual histone code specifies the binding of heterochromatin protein Rhino to a subset of piRNA source loci. 2024.01.11.575256. https://www.biorxiv.org/content/10.1101/2024.01.11.575256v1 (Accessed March 20, 2024).

Guruharsha KG, Rual JF, Zhai B, Mintseris J, Vaidya P, Vaidya N, Beekman C, Wong C, Rhee DY, Cenaj O, et al. 2011. A protein complex network of Drosophila melanogaster. *Cell* **147**: 690–703.

Ma X, Han Y, Song X, Do T, Yang Z, Ni J, Xie T. 2016. DNA damage-induced Lok/CHK2 activation compromises germline stem cell self-renewal and lineage differentiation. *Development* **143**: 4312–4323.

Parhad SS, Tu S, Weng Z, Theurkauf WE. 2017. Adaptive Evolution Leads to Cross-Species Incompatibility in the piRNA Transposon Silencing Machinery. *Developmental Cell* **43**: 60-70.e5.

Rangan P, Malone CD, Navarro C, Newbold SP, Hayes PS, Sachidanandam R, Hannon GJ, Lehmann R. 2011. piRNA Production Requires Heterochromatin Formation in Drosophila. *Current Biology* **21**: 1373–1379.

Schwarz F, Wierzbicki F, Senti K-A, Kofler R. 2021. Tirant Stealthily Invaded Natural Drosophila melanogaster Populations during the Last Century. *Molecular Biology and Evolution* **38**: 1482–1497.

Tang H-W, Spirohn K, Hu Y, Hao T, Kovács IA, Gao Y, Binari R, Yang-Zhou D, Wan KH, Bader JS, et al. 2023. Next-generation large-scale binary protein interaction network for Drosophila melanogaster. *Nat Commun* **14**: 2162.

Wang L, Barbash DA, Kelleher ES. 2020. Adaptive evolution among cytoplasmic piRNA proteins leads to decreased genomic auto-immunity. *PLOS Genetics* **16**: e1008861.

**Reviewer #3 (Fu Yang, signs review):** The authors have addressed all my questions, and I have no further questions.

We thank the reviewer for their constructive feedback and the positive evaluation of the revised manuscript.
